# The sodium/myo-inositol co-transporter SLC5A3 promotes non-small cell lung cancer cell growth

**DOI:** 10.1038/s41419-022-05017-y

**Published:** 2022-06-27

**Authors:** Zihan Cui, Chuanyong Mu, Zhichao Wu, Shu Pan, Zewen Cheng, Zhi-qing Zhang, Jun Zhao, Chun Xu

**Affiliations:** 1 Department of Cardio-Thoracic Surgery, the First Affiliated Hospital of Soochow University, Suzhou, China; 2 Department of Thoracic Surgery, Changshu Hospital Affiliated to Nanjing University of Chinese Medicine, Suzhou, China; 3 Department of Respiratory and Critical Care Medicine, The First Affiliated Hospital of Soochow University, Suzhou, China; 4 Clinical Research Center of Neurological Disease, The Second Affiliated Hospital of Soochow University, Suzhou, China

**Keywords:** Non-small-cell lung cancer, Targeted therapies

## Abstract

Identification of novel molecular signaling targets for non-small cell lung cancer (NSCLC) is important. The present study examined expression, functions and possible underlying mechanisms of the sodium/myo-inositol co-transporter SLC5A3 in NSCLC. The Cancer Genome Atlas (TCGA) database and local NSCLC tissue results demonstrated that SLC5A3 expression in NSCLC tissues (including patient-derived primary NSCLC cells) was significantly higher than that in normal lung tissues and lung epithelial cells. In primary NSCLC cells and immortalized lines, SLC5A3 depletion, using small hairpin RNA (shRNA) and CRSIRP/Cas9 methods, robustly impeded cell proliferation and migration, simultaneously provoking cell cycle arrest and apoptosis. Conversely, ectopic overexpression of SLC5A3 further enhanced proliferation and migration in primary NSCLC cells. The intracellular myo-inositol contents and Akt-mTOR activation were largely inhibited by SLC5A3 silencing or knockout (KO), but were augmented following SLC5A3 overexpression in primary NSCLC cells. Significantly, SLC5A3 KO-induced anti-NSCLC cell activity was largely ameliorated by exogenously adding myo-inositol or by a constitutively-active Akt construct. By employing the patient-derived xenograft (PDX) model, we found that the growth of subcutaneous NSCLC xenografts in nude mice was largely inhibited by intratumoral injection SLC5A3 shRNA adeno-associated virus (AAV). SLC5A3 silencing, myo-inositol depletion, Akt-mTOR inactivation and apoptosis induction were detected in SLC5A3 shRNA virus-injected NSCLC xenograft tissues. Together, elevated SLC5A3 promotes NSCLC cell growth possibly by maintaining myo-inositol contents and promoting Akt-mTOR activation.

## Introduction

Lung cancer (lung carcinoma) includes the two main types, small cell lung cancer (SCLC) and non-small cell lung cancer (NSCLC) [1, 2]. It is a primary malignancy of the respiratory system [1, 2]. Lung cancer is the leading cause of cancer-related human mortalities, affecting over 2.2 million people and caused over 1.8 million deaths around the world each year [3, 4]. It is estimated that 85% of cases of lung cancer are associated with long-term smoking of tobacco [1, 2]. NSCLC, accounting for 80–85% of all lung cancer, has two main subtypes, lung adenocarcinoma and lung squamous cell carcinoma [1, 2].

Over the past decade, the prognosis of NSCLC patients has been improved due to early cancer screening and detection, application of neoadjuvant therapies and reduced use of tobaccos [5, 6]. However, NSCLC is still considered as a primary public health threat, with the 5-year overall survival rate close to 20% [3, 4]. Different groups including ours are focusing on exploring novel and specific biomarkers as well as the promising therapeutic targets and signaling proteins for early diagnosis and efficient treatment of NSCLC [5, 6].

Using an optimized CRISPR (clustered regularly interspaced short palindromic repeats) screening technique in orthotopic xenograft models, Wei et al., recently discovered SLC5A3 (solute carrier family 5 member 3), a sodium/myo-inositol co-transporter, as a top-ranked gene essential for the tumorigenesis and progression of acute myeloid leukemia (AML) [7]. *SLC5A3* gene, located at chromosome 21q22.1, is expressed in different human tissues including brain, kidney, and lung [8]. Overexpression of SLC5A3 is likely associated with the pathophysiology of Down syndrome [8]. De Paepe *et al*., have proposed that SLC5A3 is a pathological hallmark that is upregulated in sporadic inclusion body myositis (IBM) tissues, which could be linked to the degenerative changes and inflammation of the disease [9]. Andronic et al., reported that swelling-mediated activation and upregulation of SLC5A3 promoted myo-inositol transport and regulated hypotonic volume in mammalian cells [10].

Wei et al., proposed myo-inositol as a nutrient dependency of AML and SLC5A3 transported myo-inositol to support growth of AML cells, where ISYNA1 (inositol-3-phosphate synthase 1), the rate-limiting enzyme of myo-inositol biosynthesis, was transcriptionally silenced [7]. Conversely, CRISPR/Cas9-induced knockout (KO) of SLC5A3 impeded AML cell growth in vitro and patient-derived-xenograft growth in mice [7]. Zhang et al., have recently shown that SLC5A3 is upregulated in liver cancer tissues than in the peritumoral tissues, which could be associated with increased O-GlcNAcylation and YAP expression [11]. The present study examined expression, functions and possible underlying mechanisms of SLC5A3 in NSCLC. Our results showed that SLC5A3 overexpression promoted NSCLC cell growth possibly by maintaining myo-inositol contents and promoting Akt-mTOR (mammalian target of rapamycin) activation.

## Materials and methods

### Reagents

The anti-SLC5A3 antibody (ab110368) and the anti-SLC5A11 antibody (ab55936) were obtained from Abcam (Shanghai, China). Antibodies of p-Akt (Ser 473, #9271), Akt1 (#2938), Erk1/2 (#4695), p-Erk1/2 (9101), p70 S6 Kinase (S6K1 #9202), p-S6K1 (Thr389, #9205) and cleaved caspase antibody sampler Kit (#9929) were purchased from Cell Signaling Technologies (Beverly, MA). The ISYNA1 antibody was provided by Thermo-Fisher (# PA5-44105). The primary antibodies were utilized at 1:1000–2000 and the second antibodies were utilized at 1:5000–1:10,000. Other chemicals, fluorescence dyes, reagents and antibodies were described in our previous study [12].

### Cell culture

Culturing of primary human NSCLC cells, pCan-1, pCan-2 and pCan-3, derived from three different patients, the immortalized NSCLC cell lines, A549 and H460, as well as the primary human lung epithelial cells (“pEpi”) and the immortalized BEAS-2B epithelial cells were described in previous studies [12–14]. The written-informed consent was obtained from each enrolled patient. The protocols of this study were approved by the Ethics Committee of Soochow University, in according to the World Medical Association Declaration of Helsinki.

### Human tissues

NSCLC tumor specimens and paired adjacent normal lung specimens (1.2 cm from tumor margin) were collected from a total of thirteen (*n* = 13) written-informed consent NSCLC patients administrated at the First Affiliated Hospital of Soochow University. The patients were all male, 46–77 year old, with stage-III NSCLC. The fresh tissue specimens were stored in liquid nitrogen immediately after surgery. All the administrated patients underwent standard treatments before surgery. The protocols of tissue immunohistochemistry (IHC) were described previously [15, 16].

### SLC5A3 shRNA or overexpression

Two different SLC5A3 shRNAs, shSLC5A3-S1 and shSLC5A3-S2 (containing non-overlapping sequences [17]), or the SLC5A3-expressing cDNA sequence [8], were individually sub-cloned into a GV248 construct. The shRNA construct or the overexpression construct was then transfected to HEK-293T cells along with the described lentivirus package constructs [12]. The generated lentivirus was added to cultured cells maintained in the polybrene-containing medium. Puromycin was added to select stable cells, and SLC5A3 expression was always examined. Silencing of ISYNA1 in NSCLC cells by a lentiviral ISYNA1 shRNA was through the same procedure. For in vivo studies, the shSLC5A3-S1 sequence was sub-cloned into the adeno-associated virus (AAV) construct 9 (AAV9) [12]. The construct was transfected to HEK-293 cells, generating SLC5A3 shRNA-expressing AAV. AAV was then filtered, enriched and injected to NSCLC xenografts.

### SLC5A3 knockout (KO)

The CRISPR/Cas9-SLC5A3-KO construct was generated by Genechem (Shanghai, China) and was transduced to Cas9 (from Genechem)-expressing primary NSCLC cells. The cells were then distributed to 96-well plates and subject to *SLC5A3* KO screening using qPCR assays. The single stable SLC5A3 KO cells, or koSLC5A3 cells, were established. The CRISPR/Cas9 control construct (“Cas9-C” [12]) was transduced to the control cells.

### Constitutively-active mutant Akt1

The constitutively-active Akt1 (caAkt1, S473D)-expressing adenoviral construct was from Dr. Xu’s group [18]. It was transduced to NSCLC cells and stable cells were established after selection [18].

### The quantitative real-time PCR (qRT-PCR) assays

Total RNA was extract by the TRIzol reagents (Invitrogen; Thermo-Fisher Scientific, Shanghai, China), which was reversely transcripted to cDNA by the PrimeScript RT reagent kit (Takara Bio, Japan). qRT-PCR assays were carried out by the SYBR Green PrimeScript PLUS RT-PCR kit (Takara Bio) under the on ABI Prism 7900 HT Fast Detection System (Applied Biosystems, Shanghai, China). *GAPDH* was tested as the reference gene and internal control. Data quantification was described previously [12]. Primers for *SLC5A3*, *ISYNA1*, and *GAPDH* were described previously [17, 19].

### Cell counting kit-8(CCK-8) viability assay

Cells with the applied treatments were initially seeded into 96-well plates at 5 × 10^3^ per well and cultivated for designated time periods. Afterwards, the CCK-8 solution was added into each well for 2 h. The CCK-8 optical density (OD) value in each well was determined at 450 nm.

### TUNEL (TdT-mediated dUTP nick-end labeling) staining

Cells with the designated treatments were plated in 24-well plates on coverslips and cultured for 96 h. Cells were were subject to 4% formaldehyde-PBS fixation for 15 min at room temperature and were permeabilized with 0.2% Triton X-100 in PBS for another 10 min. Cell nuclei were stained with TUNEL and DAPI. Fluorescence images were captured from five random views using a Nikon Eclipse Ti-E fluorescence microscope (Nikon Corporation, Japan).

### Flow cytometry (FACS)

Cells with designated treatments were harvested, washed, and stained with Annexin V-FITC and propidium iodide (PI) (Sigma-Aldrich), according to the manufacturer’s protocols. Cells were then analyzed by the BD FACSCalibur flow cytometer (BD Biosciences) using a Cell Quest software (version 5.0; BD Biosciences). For analyzing cell cycle distribution, cells were only stained with PI.

### Western blotting

Cells or tissues were lysed in RIPA buffer (BioVision, Shanghai, China) solution with protease inhibitor and phosphatase inhibitor cocktail (Sigma-Aldrich). Cell lysates were collected and quantified. Total proteins (25–40 µg per condition) were separated via 10–12.5% SDS-PAGE, and subsequently transferred to a PVDF membrane (EMD Millipore). Then, the membrane was blocked in 10% milk PBST solution (for 45 min) and incubated at 4 °C overnight with the applied primary antibody. Then, the membrane was incubated with HRP-conjugated secondary antibody. HRP signaling was detected by an ECL kit (Sigma). The uncropped blotting images were listed in Fig. S1.

### Migration and invasion assays

“Transwell” chambers were provided by Costar (Cambridge, MA). For each insert, 2 × 10^4^ cells in serum-free medium were placed on the upper chamber. Whereas 600 μL of 15% FBS-containing complete medium was placed in the bottom chamber. Cells were allowed to migrate for 36 h. The non-migrated cells on the upper surface were removed carefully. The migrated cells on the lower surface were then fixed, stained with crystal violet and photographed. For in vitro cell invasion assays, each chamber was coated with Matrigel (1:100 dilution; BD, Biosciences), other steps were the same.

### Other cellular functional studies

Including the nuclear EdU staining, caspase-3 activity assay, Trypan blue staining assaying of cell death, mitochondrial membrane potential (MMP) detection by JC-1 staining and single strand DNA (ssDNA) ELISA were described in detail in our previous study [12]. The level of myo-inositol was detected by a myo-inositol assay kit (ab252896, abcam, Shanghai, China) using the manufacturer’s protocol.

### Xenograft studies

The nude mice were described early [12]. Briefly, 7 × 10^6^ pCan-1 primary NSCLC cells in 0.2 mL DMEM-Matrigel solution were inoculated subcutaneously into the flank of each nude mouse. After 18 days, mice xenografts were established with volume of each tumor close to 100 mm^3^. The tumor-bearing nude mice were then randomly assigned into two groups: Ten mice in the treatment group were intratumorally injected with SLC5A3 shRNA AAV and the other ten mice in the control group received control shRNA AAV. Mice were injected with AAV daily for 10 consecutive days, 5 μL AAV each time. Tumor volume was assessed using a slide caliper every 5 days and calculated using the described formula [12]. 35 days after virus injection, all mice were anaesthetized, euthanized by cervical dislocation, and tumors were removed and measured. All animal studies were approved by the IACUC and Institute Animal Ethics Review Board of Soochow University.

### Statistical analyses

The investigator was blinded to the group allocation during the in vitro experiments. All data were with normal distribution and were presented as the mean ± standard deviation (SD). An unpaired Student’s *t* test (for comparison between two groups, EXCEL 2007) or one-way ANOVA followed by Tukey’s post hoc test (for comparison between multiple groups, SPSS23.0) was applied to evaluate statistical differences among treatment groups. *P* values < 0.05 were considered to indicate statistically significant differences.

## Results

### SLC5A3 upregulation in NSCLC

**Fig. 1:**
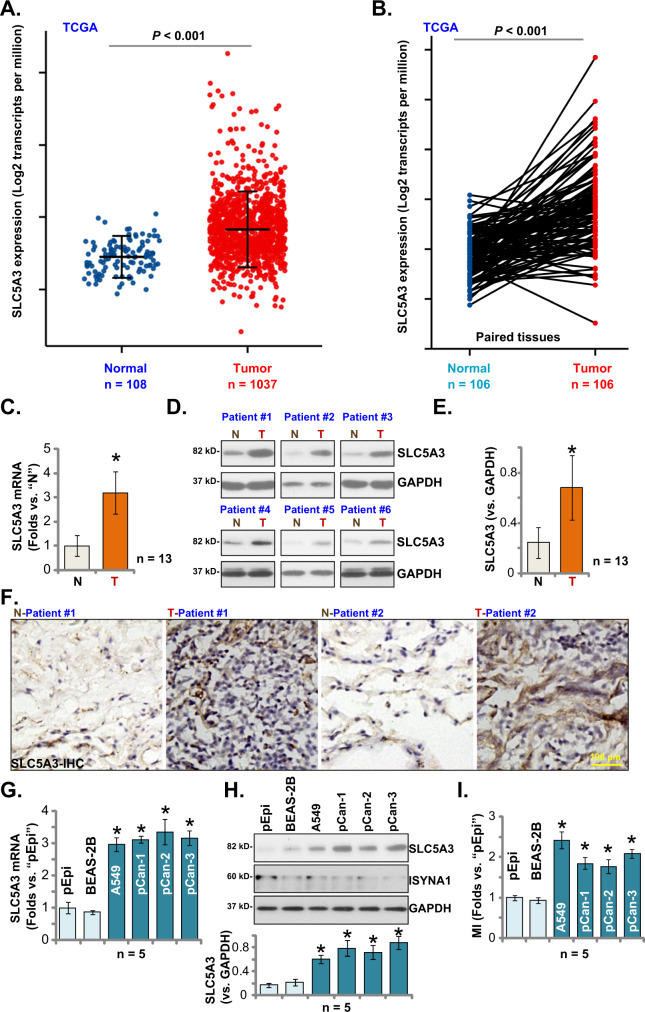
SLC5A3 upregulation in NSCLC. *SLC5A3* mRNA expression in NSCLC tumor tissues (“Tumor”, *n* = 1037) and normal lung tissues (“Normal”, *n* = 106) from TCGA cohorts was presented (**A**). *SLC5A3* mRNA expression in NSCLC tumor tissues (“Tumor”, *n* = 106) and paired adjacent normal lung tissues (“Normal”, *n* = 106) from TCGA cohorts was shown (**B**). mRNA and protein expression of SLC5A3 in local NSCLC tumor tissues (“T”, *n* = 13) and paired adjacent normal lung tissues (“N”, *n* = 13) was tested by qRT-PCR (**C**) and Western blotting (**D** and **E**) assays; The representative tissue IHC (immunohistochemistry) results confirmed SLC5A3 upregulation in NSCLC tumor slides of two representative patients (Patient #1 and Patient #2) (**F**). *SLC5A3* mRNA and listed proteins in the described NSCLC cells and epithelial cells were tested (**G** and **H**). The relative myo-inositol (MI) contents were measured as well (**I**). Data were presented as mean ± standard deviation (SD). *P < 0.05 versus “N” tissues (**C** and **E**) or “pEpi” cells (**G**–**I**). Scale bar = 100 μm (**F**).

TCGA (The Cancer Genome Atlas) database was consulted (by searching the public domain https://portal.gdc.cancer.gov) to examine *SLC5A3* expression in NSCLC. As shown, the number of *SLC5A3* transcripts in NSCLC tumor tissues (“Tumor”, *n* = 1037) was significantly higher than that in the normal lung tissues (“Normal”, *n* = 108) (Fig. 1A). In addition, *SLC5A3* mRNA expression was elevated in NSCLC tumor tissues (“Tumor”, *n* = 106) (Fig. 1B), when compared to that in the paired adjacent normal lung tissues (“Normal”, *n* = 106) (Fig. 1B).

SLC5A3 expression in local NSCLC tissues was also tested. As described, a total of thirteen (*n* = 13) different NSCLC patients with tumor resection surgeries were enrolled. NSCLC tumor tissue specimens (“T”) and the paired adjacent normal lung tissue specimens (“N”) were obtained at the time of surgery. Figure 1C demonstrated that *SLC5A3* mRNA levels in the tumor tissues was significantly higher than that in the normal lung tissues. Western blotting analyses were carried out to examine SLC5A3 protein expression. Results showed SLC5A3 protein upregulation in NSCLC tumor tissues of six representative patients (from Patient #1 to Patient #6) (Fig. 1D). When combining SLC5A3 blotting assay results of all 13 pairs of human tissues, we found that SLC5A3 protein expression in the NSCLC tumor tissues was significantly higher (Fig. 1E). The IHC results further confirmed SLC5A3 upregulation in NSCLC tumor tissues (Patient #1 and Patient #2) (Fig. 1F).

The expression of SLC5A3 in different NSCLC cells was also tested. In immortalized A549 cells and primary human NSCLC cells (pCan-1, pCan-2, and pCan-3, derived from three different patients [20]), the mRNA (Fig. 1G) and protein (Fig. 1H) expression of SLC5A3 was significantly higher than that in the primary human lung epithelial cells (“pEpi”) and established BEAS-2B epithelial cells (Fig. 1G and H). ISYNA1 protein expression was relatively low in NSCLC cells as well as in pEpi and BEAS-2B epithelial cells (Fig. 1H). Its expression was even lower in the NSCLC cells. The myo-inositol (MI) levels were however significantly elevated in the tested NSCLC cells (*P* < 0.05 versus lung epithelial cells, Fig. 1I). These results together confirmed SLC5A3 upregulation in human NSCLC tissues and cells.

### SLC5A3 depletion leads to robust anti-tumorigenic activity in NSCLC cells

**Fig. 2:**
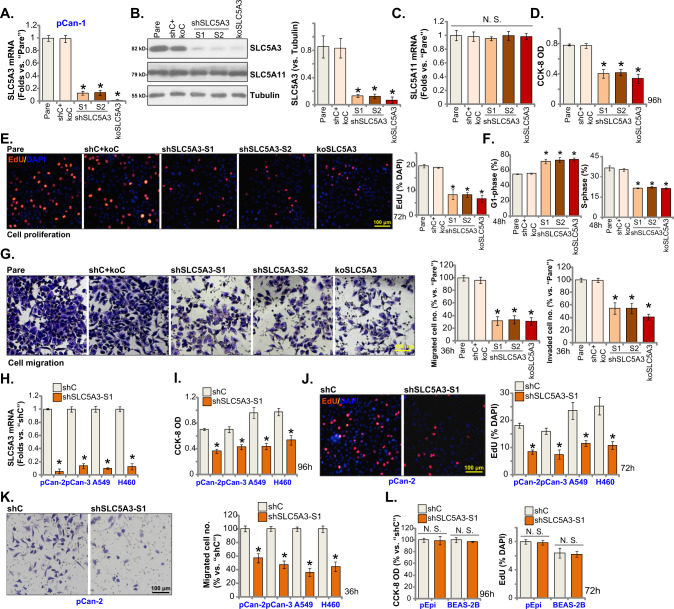
SLC5A3 depletion leads to robust anti-tumorigenic activity in NSCLC cells. Patient-derived pCan-1 primary NSCLC cells, bearing the designated SLC5A3 lentiviral shRNA (shSLC5A3-S1 and shSLC5A3-S2, with two non-overlapping sequences) or the lenti-CRISPR/Cas9-SLC5A3-KO construct (koSLC5A3), were established; The control cells were with the scramble non-sense lentiviral shRNA plus the lenti-CRISPR/Cas9-KO empty vector (“shC+koC”), expression of listed mRNAs and proteins was shown (**A**–**C**); After culturing for the designated hours, CCK-8 viability (**D**), cell proliferation (testing the ratio of EdU positively stained nuclei, (**E**), quantified G1-S cell cycle percentages (**F**), as well as cell migration (“Transwell” assays) and invasion (“Transwell assays”) (**G**) were tested by the described assays. Patient-derived primary NSCLC cells (pCan-2/3, derived from two other patients), the immortalized NSCLC lines (A549 and H460), the primary lung epithelial cells (“pEpi”) or the immortalized BEAS-2B cells, stably expressing SLC5A3 shRNA (shSLC5A3-S1) or the scramble non-sense lentiviral shRNA (shC) were established; Expression of *SLC5A3* mRNA was shown (**H**); After culturing for the designated hours, CCK-8 viability (**I** and **L**), cell proliferation (**J** and **L**), and cell migration (**K**) were tested. “Pare” indicated the parental control cells. Data were presented as mean ± standard deviation (SD, *n* = 5). *P < 0.05 versus “Pare”/“shC” group. “N. S.” indicated no statistical difference (*P* > 0.05). Each single experiment was repeated for five times. Scale bar = 100 μm.

Whether SLC5A3 can exert pro-tumorigenic activity in NSCLC cells was studied. A set of two different lentivirus-encoded shRNAs, shSLC5A3-S1 and shSLC5A3-S2, were utilized. Each of the shRNA targeted non-overlapping sequence against *SLC5A3* and was individually transduced to pCan-1 primary NSCLC cells. Puromycin was thereafter added to the shRNA virus-infected cells and stable cells were established. Alternatively, a lenti-CRISPR/Cas9-SLC5A3-KO construct was transduced to pCan-1 primary cells. After selection and subsequent KO screening, the single and stable SLC5A3-KO pCan-1 primary cells were established. These cells were named as koSLC5A3 cells. As compared to the control cells with scramble non-sense lentiviral shRNA plus the lenti-CRISPR/Cas9-KO empty vector (“shC+koC”), mRNA and protein expression of SLC5A3 were robustly decreased in shSLC5A3-expressing pCan-1 cells and koSLC5A3 pCan-1 cells (Fig. 2A and B). Conversely, mRNA and protein expression of SLC5A11 was unchanged (Fig. 2B and C).

To examine the functional consequence of SLC5A3 depletion in NSCLC cells, we first carried out the CCK-8 viability assays. SLC5A3 shRNA or KO potently decreased CCK-8 viability OD in pCan-1 primary cells (Fig. 2D), suggesting that SLC5A3 depletion exerted cytotoxic effect to NSCLC cells. As evidenced by the significantly decreased EdU-positively stained nuclei ratio (Fig. 2E), we reported that SLC5A3 silencing or KO largely impeded pCan-1 cell proliferation (Fig. 2E). Quantification of the PI-FACS assays demonstrated that SLC5A3 silencing or KO increased the ratio of the G1-phase cells, but decreased the ratio of the S-phase cells (suggesting G1-S arrest, Fig. 2F). The in vitro cell migration and invasion were separately tested by “Transwell” and “Matrigel Transwell” assays. Results demonstrated that SLC5A3 depletion, using shRNA and CRISPR/Cas9 methods, potently inhibited pCan-1 cell in vitro migration and invasion (Fig. 2G).

Next experiments were carried out to examine the potential function of SLC5A3 in other NSCLC cells. Both the primary human NSCLC cells (pCan-2/3, derived from two other human patients) or the immortalized cell lines (A549 and H460) were infected with shSLC5A3-S1-encoding lentivirus. After puromycin selection stable cells were established. qRT-PCR assays testing expression of *SLC5A3* mRNA showed that the applied shRNA resulted in robust *SLC5A3* mRNA downregulation in the primary and immortalized NSCLC cells (Fig. 2H). SLC5A3 shRNA robustly decreased CCK-8 viability in the NSCLC cells (Fig. 2I). Moreover, in the primary and immortalized NSCLC cells, the ratio of EdU-positively stained nuclei (Fig. 2J) and the number of migrated cells (“Transwell” assays, Fig. 2K) were robustly decreased after SLC5A3 silencing. On the contrast, in pEpi primary cells and established BEAS-2B cells, SLC5A3 silencing by shSLC5A3-S1 virus failed to affect CCK-8 cell viability and proliferation (by measuring the EdU-positive nuclei ratio) (Fig. 2L). These results together demonstrated that SLC5A3 silencing/KO led to robust anti-tumorigenic activity in NSCLC cells and inhibited cell viability, proliferation and mobility.

### SLC5A3 depletion provokes apoptotic death in NSCLC cells

**Fig. 3:**
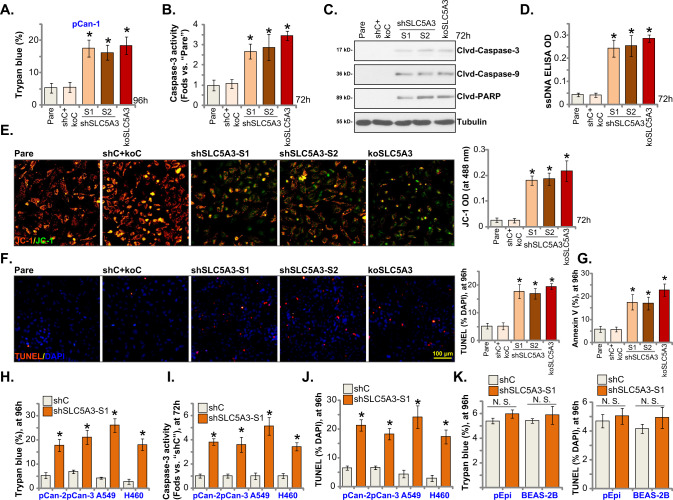
SLC5A3 depletion provokes apoptotic death in NSCLC cells. Patient-derived pCan-1 primary NSCLC cells, bearing the designated SLC5A3 lentiviral shRNA (shSLC5A3-S1 and shSLC5A3-S2) or the lenti-CRISPR/Cas9-SLC5A3-KO construct (koSLC5A3), were established; The control cells were with the scramble non-sense lentiviral shRNA plus the lenti-CRISPR/Cas9-KO empty vector (“shC+koC”). After culturing for the designated hours, cell death (Trypan blue ratio, **A**), the relative caspase-3 activity (**B**), expression of apoptosis-related proteins (Western blotting assays, **C**), ssDNA contents (ELISA OD, **D**) and mitochondrial depolarization (JC-1 green monomers accumulation, **E**) were tested. Cell apoptosis was examined by measuring the ratio of TUNEL-positively stained nuclei (**F**) and the number of Annexin V-positive cells (FACS assays, **G**). Patient-derived primary NSCLC cells (pCan-2/3, derived from two other patients), the immortalized NSCLC lines (A549 and H460), the primary lung epithelial cells (“pEpi”) or the immortalized BEAS-2B cells, stably expressing the SLC5A3 shRNA (shSLC5A3-S1) or the scramble non-sense lentiviral shRNA (shC) were established. After culturing for the designated hours, cell death (by measuring Trypan blue ratio, **H** and **K**), the relative caspase-3 activity (**I**), and apoptosis (by measuring the percentage of the TUNEL-positive nuclei, **J** and **K**) were tested similarly. “Pare” indicated the parental control cells. Data were presented as mean ± standard deviation (SD, *n* = 5). *P < 0.05 versus “Pare”/“shC” group. “N. S.” indicated no statistical difference (*P* > 0.05). Each single experiment was repeated for five times. Scale bar = 100 μm.

Since SLC5A3 depletion exerted robust anti-tumorigenic activity in NSCLC cells, we next tested whether apoptosis was induced. As evidenced by the increased Trypan blue-positive staining cells, we showed that SLC5A3 silencing, by shSLC5A3-S1 and shSLC5A3-S2, or KO induced significant pCan-1 primary cell death (Fig. 3A). The relative caspase-3 activity was robustly augmented in stable pCan-1 cells with the SLC5A3 shRNA or the CRISPR/Cas9-SLC5A-KO construct (Fig. 3B). Furthermore, SLC5A3 silencing or KO induced cleavages of caspase-3, poly (ADP-ribose) polymerase (PARP) and caspase-9 in pCan-1 primary cells (Fig. 3C). Increased DNA breaks were observed following SLC5A3 silencing or KO, as the ssDNA contents were accumulated (Fig. 3D). SLC5A3 shRNA or KO induced collapse of MMP and provoked mitochondrial depolarization, which was evidenced by the accumulation of JC-1 green monomers in the mitochondria of pCan-1 primary cells (Fig. 3E). To support apoptosis activation, we found that the ratio of TUNEL-positively stained nuclei was significantly augmented in pCan-1 primary cells with SLC5A3 shRNA or KO (Fig. 3F). Moreover, FACS assay results (Fig. 3G) showed that the number of pCan-1 cells with positive Annexin V staining was dramatically increased after SLC5A3 silencing/depletion. The control treatment, shC+koC, failed to significantly provoke caspase-apoptosis activation in the pCan-1 primary cells (Fig. 3A–G).

Treatment with the SLC5A3 inhibitor phlorizin [21, 22] largely inhibited proliferation (EdU-nuclei ratio decreasing, Fig. S2A) and migration (Fig. S2B) in pCan-1 primary NSCLC cells. Moreover, the SLC5A3 inhibitor induced mitochondrial depolarization (Fig. S2C) and apoptosis (TUNEL-positive nuclei increasing, S2D) in pCan-1 cells.

In other primary human NSCLC cells (pCan-2/3) and the immortalized cell lines (A549 and H460), SLC5A3 silencing by the lentiviral shSLC5A3-S1 induced significant cell death (Trypan blue staining increase, Fig. 3H). In addition, shSLC5A3-S1 increased the relative caspase-3 activity (Fig. 3I). The ratio of TUNEL-positively stained nuclei was significantly augmented in SLC5A3-silenced NSCLC cells, supporting apoptosis activation (Fig. 3J). In the primary human lung epithelial cells (“pEpi”) and BEAS-2B cells, SLC5A3 silencing by shSLC5A3-S1 failed to provoke significant cell death (Fig. 3K) and apoptosis (Fig. 3K). Collectively, SLC5A3 silencing or KO provoked apoptotic death in NSCLC cells.

### SLC5A3 overexpression exerts pro-tumorigenic activity in NSCLC cells

**Fig. 4:**
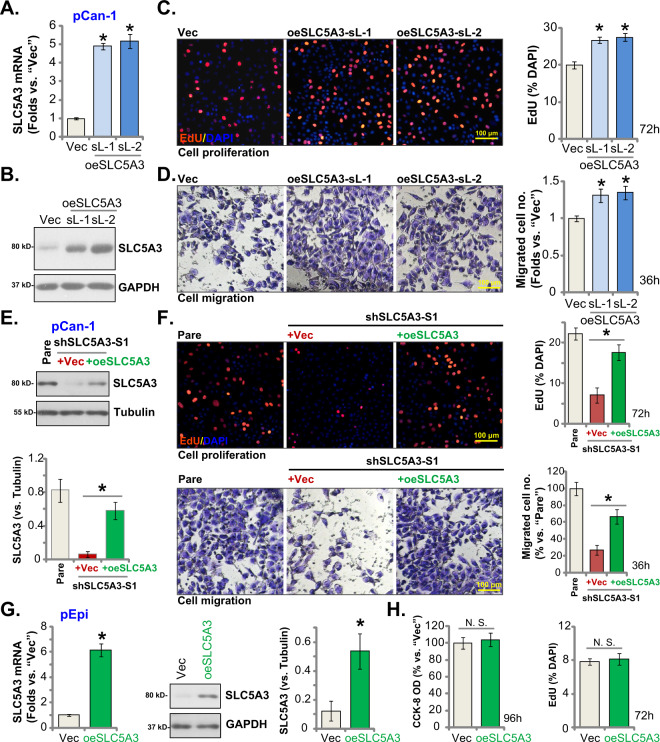
SLC5A3 overexpression exerts pro-tumorigenic activity in NSCLC cells. Patient-derived pCan-1 primary NSCLC cells, bearing SLC5A3-expressing lentiviral construct (oeSLC5A3-sL-1 and oeSLC5A3-sL-2, two different selections) were established, control cells were with the empty vector (“Vec”); *SLC5A3* mRNA and protein expression was tested (**A** and **B**). After culturing for the designated hours, cell proliferation (**C**) and migration (**D**) were examined. The pCan-1 primary NSCLC cells stably expressing shSLC5A3-S1 were further transduced with the SLC5A3-expressing lentiviral construct (“+oeSLC5A3”) or the empty vector, expression of listed proteins was shown (**E**); After culturing for the designated hours, cell proliferation (**F**) and migration (**F**) were tested. The primary lung epithelial cells (“pEpi”), bearing the SLC5A3-expressing lentiviral construct (oeSLC5A3) or the empty vector (“Vec”), were established; *SLC5A3* mRNA and protein expression was shown (**G**); After culturing for the designated hours, CCK-8 viability (**H**), cell proliferation (testing the percentage of EdU positively-stained nuclei, **H**), were examined. Data were presented as mean ± standard deviation (SD, *n* = 5). *P < 0.05 versus “Vec” group. *P < 0.05 (**F**). “N. S.” indicated no statistical difference (*P* > 0.05). Each single experiment was repeated for five times. Scale bar = 100 μm.

Next, we hypothesized that ectopic overexpression of SLC5A3 should exert opposite effect and induce pro-tumorigenic activity in NSCLC cells. Therefore, pCan-1 primary cells were infected with the SLC5A3-expressing lentivirus, and puromycin was added to select stable cells: oeSLC5A3-sL-1 and oeSLC5A3-sL-2 (two selections). To confirm SLC5A3 overexpression, we showed that expression of *SLC5A3* mRNA increased over five–six folds in oeSLC5A3 pCan-1 cells (Fig. 4A). Moreover, SLC5A3 protein elevation was detected in the oeSLC5A3-sL-1 and oeSLC5A3-sL-2 cells (Fig. 4B). Ectopic overexpression of SLC5A3 increased the ratio of EdU-positively stained nuclei in pCan-1 primary cells, suggesting that SLC5A3 overexpression accelerated proliferation (Fig. 4D). Results from the “Transwell” assays further showed that pCan-1 cell migration was augmented following ectopic SLC5A3 overexpression (Fig. 4E). These results supported that ectopic SLC5A3 overexpression exerted pro-tumorigenic activity in NSCLC cells.

The SLC5A3-expressing lentiviral construct was also transduced to the shSLC5A3-S1-expressing pCan-1 NSCLC cells and restored SLC5A3 expression (“+oeSLC5A3”, Fig. 4E). SLC5A3 re-expression partially rescued cell proliferation and migration in the koSLC5A3 pNSCLC-1 cells (Fig. 4F). These results further supported the key role of SLC5A3 in pCan-1 cell growth. Conversely, in the primary lung epithelial cells (“pEpi”), ectopic overexpression of SLC5A3 (Fig. 4G), using the described construct, failed to significantly increase CCK-8 viability and proliferation (Fig. 4H), again supporting a cancer cell specific effect by SLC5A3.

### SLC5A3 is important for maintaining the intracellular myo-inositol level in NSCLC cells

**Fig. 5:**
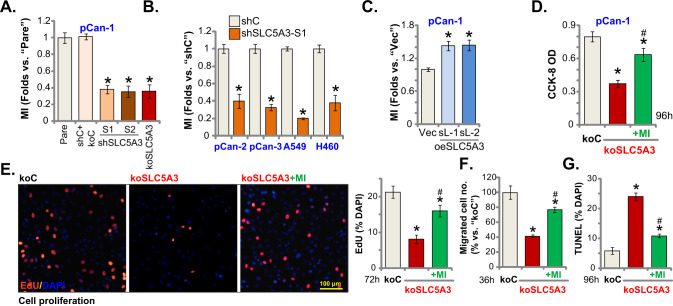
SLC5A3 is important for maintaining the intracellular myo-inositol level in NSCLC cells. The described NSCLC cells, bearing the designated SLC5A3 lentiviral shRNA (shSLC5A3-S1/shSLC5A3-S2), the lenti-CRISPR/Cas9-SLC5A3-KO construct (koSLC5A3), the scramble non-sense lentiviral shRNA (“shC”) or plus the lenti-CRISPR/Cas9-KO empty vector (“shC+koC”), the SLC5A3-expressing lentiviral construct (oeSLC5A3-sL-1 and oeSLC5A3-sL-2, two different selections) or the empty vector (“Vec”) were established, and the cellular myo-inositol (“MI”) levels were examined (**A**–**C**). The koSLC5A3 pCan-1 primary NSCLC cells were treated with or without myo-inositol (2.5 mM) and cultured for designated time periods, the control cells were with the lenti-CRISPR/Cas9-KO empty vector (“koC”); The cell viability, proliferation, in vitro cell migration and apoptosis were examined by CCK-8 (**D**), nuclear EdU staining (**E**), “Transwell” (**F**) and nuclear TUNEL staining (**G**) assays, respectively, and results were quantified. “Pare” indicated the parental control NSCLC cells. Data were presented as mean ± standard deviation (SD, *n* = 5). *P < 0.05 versus “Pare”/“shC”/“Vec”/“koC” group. ^#^*P* < 0.05 versus “koSLC5A3” group (**D**–**G**). Each single experiment was repeated for five times. Scale bar = 100 μm.

SLC5A3 is essential for the maintaining and transporting myo-inositol [9, 23, 24]. We found that SLC5A3 silencing by shRNA (shSLC5A3-S1 or shSLC5A3-S2) or SLC5A3 KO induced robust myo-inositol depletion in pCan-1 primary NSCLC cells (Fig. 5A). In shSLC5A3-S1-expressing pCan-1 cells, silencing ISYNA1 by the lentiviral shRNA (Fig. S2E) only slightly decreased myo-inositol levels (Fig. S2F). These results supported that SLC5A3 should be the primary mechanism for maintaining myo-inositol levels in NSCLC cells. Likewise, in other primary human NSCLC cells (pCan-2/3) or immortalized cell lines (A549 and H460), shSLC5A3-S1-induced silencing of SLC5A3 led to dramatic myo-inositol depletion (Fig. 5B). In contrast, the intracellular myo-inositol contents were augmented in SLC5A3-overexpressed pCan-1 cells (oeSLC5A3-sL-1 and oeSLC5A3-sL-2) (Fig. 5C). To examine whether myo-inositol depletion contributed to SLC5A3 KO-induced anti-NSCLC cell activity, myo-inositol was exogenously added. As demonstrated myo-inositol (2.5 mM) largely ameliorated SLC5A3 KO-induced CCK-8 cell viability reduction (Fig. 5D), proliferation arrest (EdU-positive nuclei ratio reduction, Fig. 5E) and in vitro cell migration inhibition (Fig. 5F). Moreover, Fig. 5G demonstrated that SLC5A3 KO-induced apoptosis (TUNEL ratio increasing) was alleviated by exogenously adding myo-inositol in pCan-1 cells.

### SLC5A3 is important for Akt-mTOR activation in NSCLC cells

**Fig. 6:**
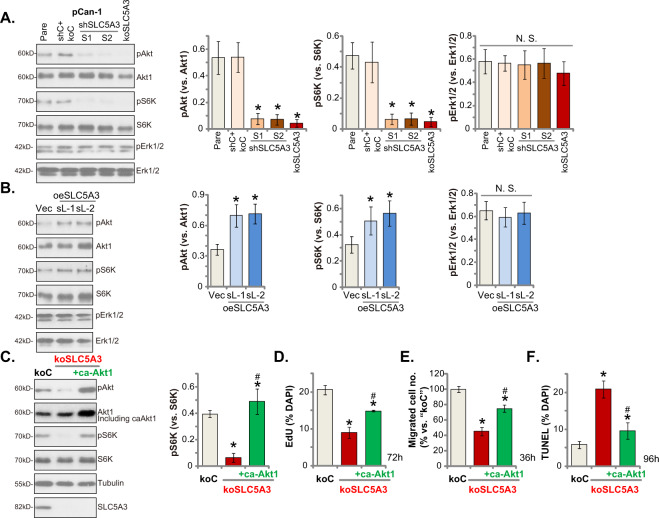
SLC5A3 is important for Akt-mTOR activation in NSCLC cells. The stable pCan-1 primary cells, bearing the designated SLC5A3 lentiviral shRNA (shSLC5A3-S1 and shSLC5A3-S2), the lenti-CRISPR/Cas9-SLC5A3-KO construct (koSLC5A3), the scramble non-sense lentiviral shRNA plus the lenti-CRISPR/Cas9-KO empty vector (“shC+koC”), the SLC5A3-expressing lentiviral construct (oeSLC5A3-sL-1 and oeSLC5A3-sL-2) or the empty vector (“Vec”) were established; Expression of listed proteins was shown (**A** and **B**). The koSLC5A3 pCan-1 primary cells were further transduced with or without the adenoviral constitutively-active Akt1 (“caAkt1”, S473D), and stable cells established after selection. Expression of listed proteins was shown (**C**); Cells were further cultured for the designated time periods, and cell proliferation (by measuring EdU-positive nuclei ratio, **D**), in vitro cell migration (**E**) and apoptosis (by measuring TUNEL-positively stained nuclei ratio, **F**) were tested by the described methods. “Pare” indicated the parental control NSCLC cells. Data were presented as mean ± standard deviation (SD, *n* = 5). *P < 0.05 versus “Pare”/“Vec”/“koC”/“DMSO” group. ^#^*P* < 0.05 versus “koSLC5A3” group (**D**–**F**). “N. S.” indicated no statistical difference (*P* > 0.05, **A** and **B**). Each single experiment was repeated for five times.

Inositol second messengers regulating cell signaling pathways are essential for the progression of cancer. Inositols were shown to induce activation of Akt [25]. Considering that hyper-activation of PI3K-Akt-mTOR cascade is vital for tumorigenesis, progression and therapy-resistance of NSCLC [6, 26–28], we therefore analyzed the potential role of SLC5A3 on Akt-mTOR activation in NSCLC cells. As shown, in stable pCan-1 primary NSCLC cells with SLC5A3 shRNA (shSLC5A3-S1, shSLC5A3-S2) or the lenti-CRISPR/Cas9-SLC5A3-KO construct (koSLC5A3), levels of phosphorylated Akt and S6K1 were significantly decreased (Fig. 6A). These results implied that SLC5A3 silencing or KO inhibited Akt-mTOR activation in NSCLC cells (Fig. 6A). Total Akt and S6K1 as well as Erk1/2 phosphorylation and expression were unchanged by SLC5A3 depletion in pCan-1 primary (Fig. 6A).

Conversely, ectopic overexpression of SLC5A3 augmented Akt-mTOR activation in NSCLC cells, as the levels of phosphorylated Akt and S6K1 were enhanced in oeSLC5A3-sL-1 and oeSLC5A3-sL-2 pCan-1 cells (Fig. 6B). Total Akt and S6K1 expression as well as Erk1/2 phosphorylation and expression were not significantly altered by SLC5A3 overexpression (Fig. 6B).

To support that SLC5A3-driven NSCLC cell progression was associated with Akt-mTOR activation, an adenoviral constitutively-active Akt1 (“caAkt1”, S473D) was transduced to koSLC5A3 pCan-1 cells, and stable cells were established after selection. As shown, caAkt1 restored Akt and S6K1 phosphorylation in SLC5A3 KO pCan-1 primary cells, without affecting SLC5A3 expression (Fig. 6C). Importantly, caAkt1 mitigated SLC5A3 KO-induced inhibitions on cell proliferation (by measuring EdU-positive nuclei ratio, Fig. 6D) and in vitro cell migration (Fig. 6E). Moreover, TUNEL staining assay results showed that apoptosis induction in koSLC5A3 pCan-1 cells was ameliorated by caAkt1 (Fig. 6F). These results supported that SLC5A3-driven NSCLC cell progression was through, at least in part, by promoting Akt-mTOR activation. In the control pCan-1 primary cells, caAkt1 increased Akt-S6K phosphorylation (Fig. S2G). Expectably, it also augmented cell proliferation (EdU assays, Fig. S2H) and migration (Fig. S2I) in pCan-1 cells.

### SLC5A3 is required for NSCLC xenograft growth in vivo

**Fig. 7:**
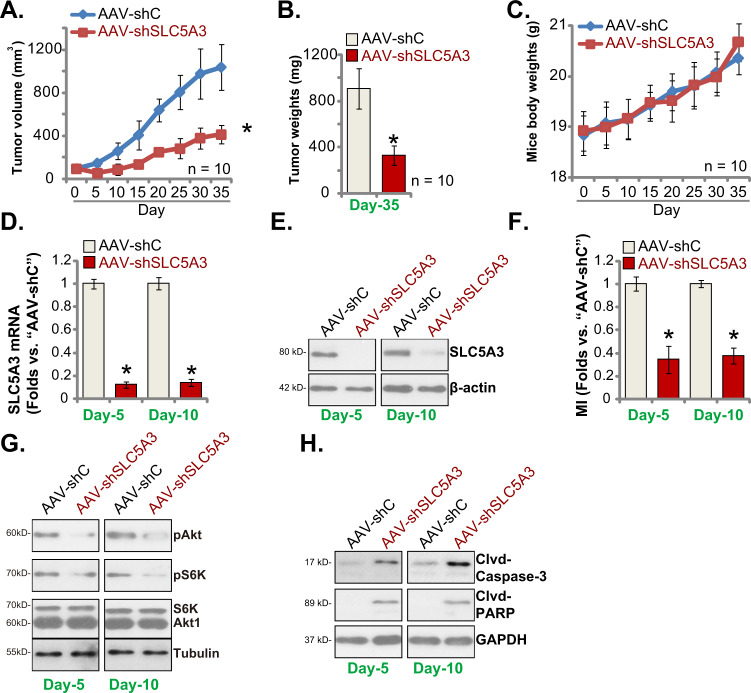
SLC5A3 is required for NSCLC xenograft growth in vivo. The pCan-1 xenograft-bearing nude mice were subject to intratumoral injection of the shSLC5A3-S1-expressing adeno-associated virus (AAV-shSLC5A3) or the scramble control shRNA adeno-associated virus (“AAV-shC”), daily for 10 days; The estimated tumor volumes (in mm^3^, **A**) and the mice body weights (in grams, **C**) were recorded every 5 days, from “Day-0” to “Day-35”. The mice were decapitated and killed at “Day-35”, and pCan-1 xenografts were weighted individually (**B**). At “Day-5” and “Day-10”, 4 h after AAV injection, one pCan-1 xenograft per group was isolated. Expression of listed genes and proteins in xenograft tumor lysates was measured (**D**, **E**, **G** and **H**); The myo-inositol (“MI”) contents were measured as well (**F**). Data were presented as mean ± standard deviation (SD). *P < 0.05 versus “AAV-shC” group. Scale bar = 100 μm (**H**).

To support that SLC5A3 is important to drive NSCLC growth in vivo, animal xenograft studies were carried out. Through subcutaneous injection, pCan-1 primary NSCLC cells (seven million cells per mouse) were inoculated into the nude mice. After 18 days of cell injection, the volume of each pCan-1 xenograft was close to 100 mm^3^ (labeled as “Day-0”). The shSLC5A3-S1-expressing adeno-associated virus (AAV-shSLC5A3, S1) or the scramble control shRNA adeno-associated virus (“AAV-shC”) were intratumorally injected to the pCan-1 xenografts. AAV injection was carried out daily for 10 consecutive days (“Day-0” to “Day-10”). The volumes of the xenografts were then recorded every 5 days, from “Day-0” to “Day-35” (Fig. 7A).

As shown AAV-shSLC5A3 injection robustly inhibited the growth of pCan-1 xenografts in nude mice (Fig. 7A). All pCan-1 xenografts were carefully removed and weighted at “Day-35”. We showed that pCan-1 xenografts with AAV-shSLC5A3 injection were significantly lighter than those of with AAV-shC injection (Fig. 7B). The mice body weight curve found no significant difference in the body weights between the two groups of animals (Fig. 7C).

Whether AAV-shSLC5A3 resulted in similar signaling change in vivo was studied. At “Day-5” and “Day-10”, four hours after AAV injection, we isolated one pCan-1 xenograft per group. The total four tumors were obtained. As shown mRNA and protein expression of SLC5A3 were dramatically decreased in AAV-shSLC5A3-injected pCan-1 xenograft tissues (Fig. 7D and E). Moreover, myo-inositol contents were dramatically decreased in pCan-1 xenograft tissues with AAV-shSLC5A3 injection (Fig. 7F). Figure 7G demonstrated that Akt and S6K1 phosphorylation was significantly inhibited in SLC5A3-silenced pCan-1 xenograft tissues. Conversely, levels of cleaved-caspsae-3 and cleaved-PARP were increased in AAV-shSLC5A3-injected pCan-1 xenografts (Fig. 7H), supporting apoptosis activation. These results were consistent with the in vitro signaling findings.

## Discussion

It is estimated that over 300 non-synonymous mutations could occur in each NSCLC, yet only a handful of them can drive tumorigenesis [29–31]. The oncogenic drivers are in several key signalling cascades, promoting cancer cell survival, growth and proliferation as well as migration, metastasis and therapy resistance [29–31]. These key drivers, including p53 and Rb inactivation as well as MYC, RAS, Akt-mTOR overactivation, are not only sufficient, but also necessary for NSCLC formation and progression [32–34]. Advances in understanding of the genomics and the molecular pathology of NSCLC should help to explore therapeutic inhibition of these targets in NSCLC [29–31].

Here we provided experimental evidences supporting that SLC5A3 could be a valuable therapeutic target of NSCLC. SLC5A3 expression is significantly elevated in NSCLC tissues and various NSCLC cells. In different patient-derived primary NSCLC cells and immortalized lines, SLC5A3 silencing (using targeted shRNAs) or KO (by the CRSIPR/Cas9 method) robustly impeded cell viability, proliferation and migration in vitro, and provoking G1-S arrest and apoptosis. No significant cytotoxicity was detected in SLC5A3-silenced normal lung epithelial cells. Conversely, ectopic overexpression of SLC5A3, by the lentiviral construct, further enhanced proliferation and migration of NSCLC cells. SLC5A3 shRNA AAV injection potently suppressed NSCLC cell growth in vivo in a PDX model. Thus, targeting SLC5A3 could be a novel therapeutic strategy against NSCLC.

Metabolic reprogramming is a key hallmark of NSCLC and is critical for the sustained cancer cell proliferation [35–37]. Like other malignancies, altered metabolic features in NSCLC can provide enough nutrients for cancer growth [35–37]. Ji et al., have demonstrated that SLC7A11 is a key regulator of metabolic reprogramming during NSCLC tumorigenesis and progression and is essential for glucose metabolism, glutamine dependency, and intracellular GSH/GSSG redox balance in NSCLC cells [38]. SLC7A11 overexpression maintained metabolic requirements and promoted NSCLC cell growth in vitro and in vivo [38]. Conversely, SLC7A11 silencing can significantly inhibited NSCLC growth [38]. Wei et al., have recently performed CRISPR-based screenings in AML cell lines and patient-derived-xenograft AML animal models [23], allowing systematic evaluation of the top AML-dependent candidate genes [23]. SLC5A3 was reported as the top-ranked gene target of AML [23]. Increased SLC5A3 expression in AML can transport myo-inositol to maintain and promote AML cell growth in vitro and in vivo [23].

In the current study, we show that SLC5A3 is essential for maintaining myo-inositol contents in NSCLC cells. The intracellular myo-inositol contents were decreased following SLC5A3 shRNA or KO in NSCLC cells, but were increased with ectopic SLC5A3 overexpression. Myo-inositol contents were also decreased in SLC5A3 shRNA virus-injected NSCLC xenograft tissues. Remarkably, SLC5A3 KO-induced inhibitions on NSCLC cell growth and proliferation were largely attenuated by exogenously adding myo-inositol. Thereby, SLC5A3 elevation in NSCLC is vital for maintaining myo-inositol contents, supporting NSCLC cell growth.

The PI3K-Akt-mTOR cascade is often hyper-activated in NSCLC, possibly due to various genetic alterations including *phosphatase and tensin homolog* (*PTEN*) depletion, *PI3KCA* mutation and sustained activation of different receptor tyrosine kinase [6, 27, 28, 39, 40]. It is a key signaling cascade and a vital therapeutic target for NSCLC [6, 27, 28, 39, 40]. PI3K-Akt-mTOR inhibition can result in significant anti-NSCLC cell activity, either alone or in combination with other strategies [27, 41–43].

In primary NSCLC cells, Akt-S6K1 phosphorylation was largely inhibited by SLC5A3 shRNA or KO, but was enhanced after SLC5A3 overexpression. In vivo, Akt-mTOR inactivation was detected in SLC5A3 shRNA virus-injected NSCLC xenograft tissues. Remarkably, SLC5A3 KO-induced anti-NSCLC cell activity was ameliorated, but not reversed, by the caAkt1. Yet, Akt-S6K1 phosphorylation in SLC5A3 KO cells was even higher than the control cells after caAkt1 treatment. Thus, other mechanisms should also participate in SLC5A3-driven NSCLC cell progression.

Indeed, we show that SLC5A3 elevation in NSCLC cells is vital for maintaining myo-inositol contents, which could be another important mechanism supporting NSCLC cell growth. Moreover, myo-inositol is a precursor for phosphatydylinostol-4,5-biphosphate (PIP2) and several other intracellular mediators, which are reportedly key signaling molecules for cancer progression [44, 45]. It will be interesting to test whether SLC5A3 are important for the production these mediators in NSCLC cells. Moreover, SLC5A3 is a crucial regulator of cell response to osmotic stress, the latter plays an important role in regulating various signaling cascades and cancer cell progression, including cell migration, proliferation and DNA repair [46–52]. Swelling-induced activation and upregulation of SLC5A3 can promote myo-inositol transport and regulate hypotonic volume [10]. Therefore, further studies will be needed to explore the possible relationship between increased SLC5A3 expression and osmotic stress response in NSCLC cells.

## Conclusion

SLC5A3 promoted NSCLC cell growth possibly by maintaining myo-inositol contents and Akt-mTOR activation. SLC5A3 could therefore be a promising and important therapeutic target of NSCLC.

## Supplementary information

Figure S1

Figure S2

checklist form

## Data Availability

All data are available upon request.
