# Supplementary material for: The sodium/myo-inositol co-transporter SLC5A3 promotes non-small cell lung cancer cell growth
Source: Cell Death Dis. 2022 Jun 27;13(6):569. doi: 10.1038/s41419-022-05017-y (PMC9237060; doi:10.1038/s41419-022-05017-y)

Figure S1. The un-cropped blotting images of the study.

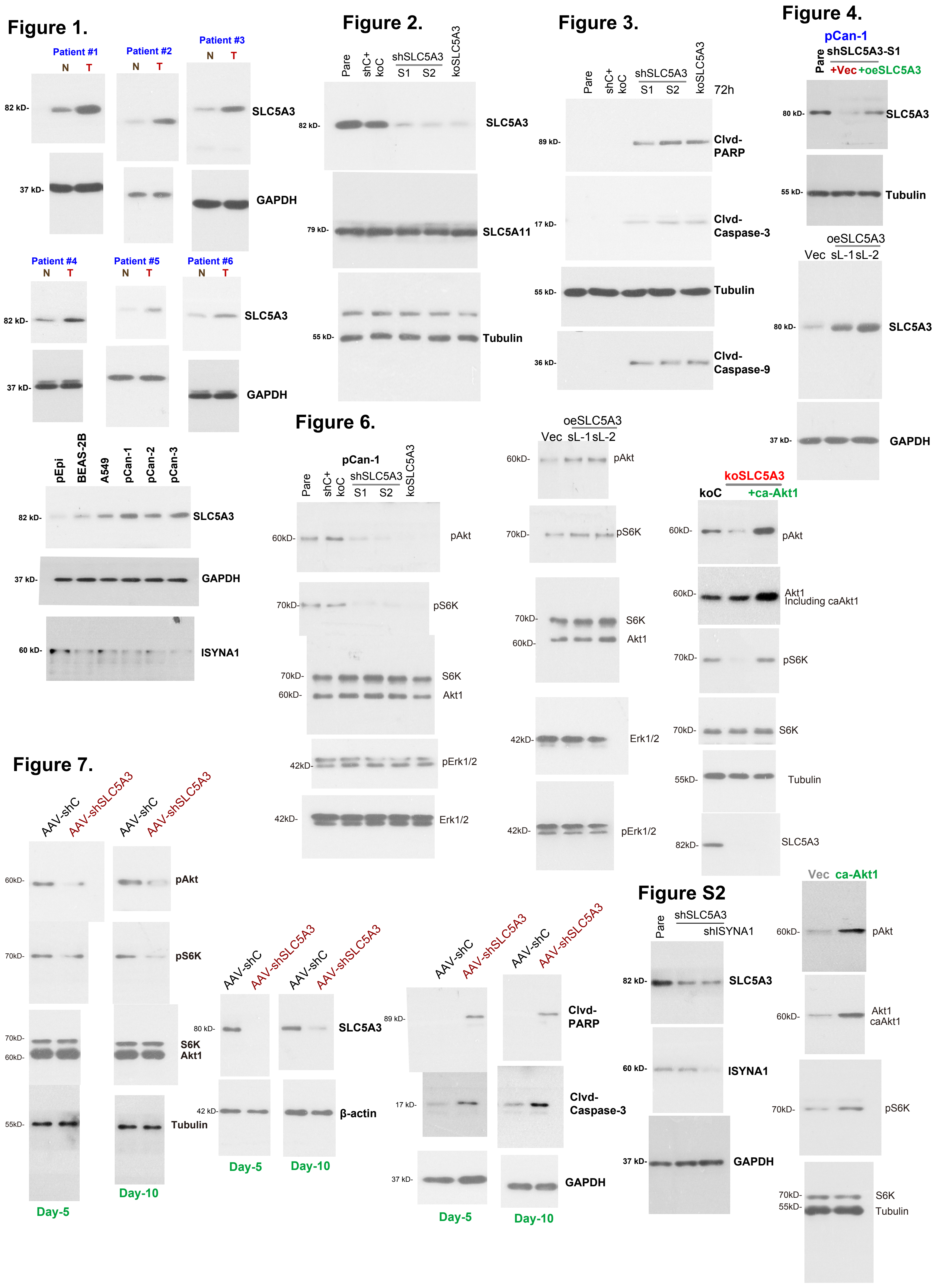

Supplement: Supplementary file 1 — Figure S1 [file 41419_2022_5017_MOESM1_ESM.pdf]
