# Supplementary material for: The sodium/myo-inositol co-transporter SLC5A3 promotes non-small cell lung cancer cell growth
Source: Cell Death Dis. 2022 Jun 27;13(6):569. doi: 10.1038/s41419-022-05017-y (PMC9237060; doi:10.1038/s41419-022-05017-y)
Supplement: Supplementary file 2 — Figure S2 [file 41419_2022_5017_MOESM2_ESM.pdf]

Figure S2

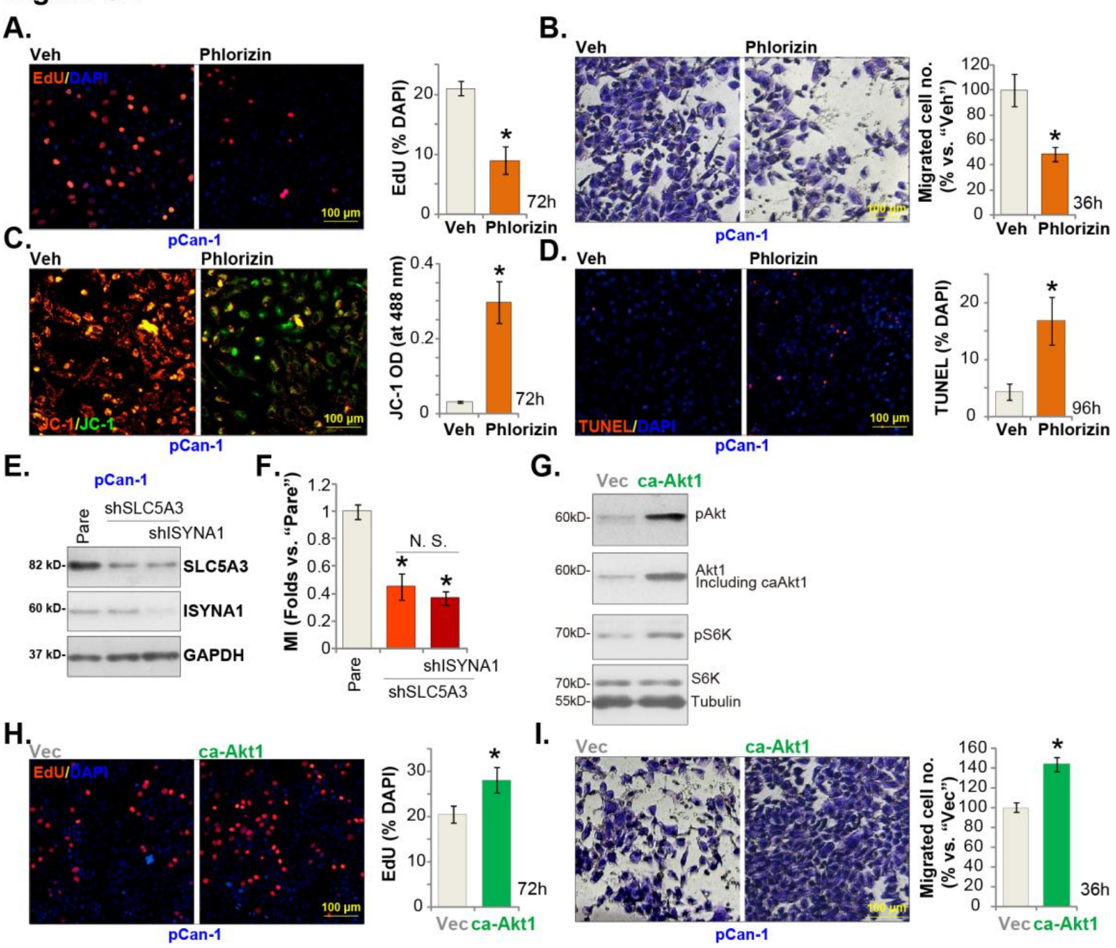

Figure S2. The pCan-1 primary NSCLC cells were treated with phlorizin (0.5 mM) or vehicle control (0.2% DMSO, "Veh"), and cultured for indicated time periods; Cell proliferation (EdU-positive nuclei ratio, **A**), migration (B), mitochondrial depolarization (JC-1 green monomers accumulation, **C**) and apoptosis (**D**, TUNEL assays) were examined. Stable pCan-1 primary NSCLC cells bearing shSLC5A3-S1 ("shSLC5A3") were further infected with lentiviral ISYNA1 shRNA ("shISYNA1"), expression of listed proteins (**E**) and cellular myo-inositol ("MI") levels (**F**) were examined. The pCan-1 primary cells were transduced with the adenoviral constitutively-active Akt1 ("caAkt1", S473D) or the empty vector ("Vec"), and stable cells established after selection. Expression of listed proteins was shown (**G**); Cells were further cultivated for the indicated time periods, cell proliferation (EdU-positive nuclei ratio, **H**) and migration (**I**) were tested. "Pare" indicated the parental control cells. Data were presented as mean ± standard deviation (SD, n=5). ****P*** < 0.05 versus "Veh"/"Pare"/"Vec" cells. "N. S." indicated no statistical difference (***P*** > 0.05). Data were presented as mean ± standard deviation (SD, n=5). Each single experiment was repeated for five times. Scale bar = 100 µm.
